# Concurrent validity of the short-form Family Impact Scale (FIS-8) in 4-year-old US children

**DOI:** 10.1186/s12887-022-03437-5

**Published:** 2022-07-04

**Authors:** W. M. Thomson, L. A. Foster Page, S. M. Levy, M. A. Keels, A. T. Hara, M. Fontana

**Affiliations:** 1 Department of Oral Sciences, Sir John Walsh Research Institute, School of Dentistry, The University of Otago, Dunedin, New Zealand; 2 Oregon Health & Science University School of Dentistry, Portland, CA USA; 3 University of Iowa, Iowa City, IA USA; 4 Duke University, Durham, NC USA; 5 Indiana University, Indianapolis, IN USA; 6 University of Michigan, Ann Arbor, MI USA

**Keywords:** Quality of Life, Child, Family, Oral Health

## Abstract

**Background:**

US data on the validity and reliability of the short-form Family Impact Scale (FIS-8; a scale for measuring the impact of a child’s oral condition on his/her family) are lacking.

**Methods:**

Cross-sectional analysis of data on four-year-old US children taking part in a multi-center cohort study. For child-caregiver dyads recruited at child age 12 months, the impact of the child’s oral condition on the family was assessed at age 48 months using the FIS-8, with a subsample of 422 caregivers (from 686 who were approached). Internal consistency reliability was assessed using Cronbach’s α, with concurrent validity assessed against a global family impact item (“How much are your family’s daily lives affected by your child’s teeth, lips, jaws or mouth?”) and a global oral health item (“How would you describe the health of your child’s teeth and mouth?”).

**Results:**

Cronbach’s alpha was 0.83. Although gradients in mean scores across ordinal response categories of the global family impact item were inconsistent, there were marked, consistent gradients across the ordinal categories of the global item on the child’s oral health, with scores highest for those rating their child’s oral health as ‘Poor’.

**Conclusions:**

While the findings provide some evidence for the utility of the FIS in a US child sample, the study’s replication in samples of preschoolers with greater disease experience would be useful.

## Background

The Family Impact Scale (FIS) is part of the suite of scales—collectively known as the Child Oral Health Quality of Life questionnaire (COHQOL)—which was developed for measuring the impact of oral conditions on children and their families [1, 2]. The 14-item FIS was designed for use with proxy informants, usually a child’s mother or father, because very young children tend to be unreliable in that respect. A short-form version (the FIS-8, comprising 8 items) was subsequently developed and shown to be valid and responsive [3]. It has three subscales, representing the domains of parental/family activity (4 items), parental emotions (2 items) and family conflict (2 items).

Although the validity and reliability of the FIS have been demonstrated in a number of studies [2–6], data from the United States (U.S.) are lacking. Only one previous U.S. study has reported using FIS data, but with the families of older children [7]. It may be that the emergence of the 13-item Early Childhood Oral Health Impact Scale (ECOHIS) in North Carolina [8] has meant that US researchers have tended to opt for the measure with US provenance. The ECOHIS arose from work with the original 45-item pool used by the team which had developed the original P-CPQ and FIS scales [1]. The ECOHIS team obtained ratings of those items from health professionals (and associated staff and researchers) experienced in dealing with young children. The 36 items remaining from that process then underwent item reduction with 30 parents of 3–5-year-old children, resulting in the 13-item ECOHIS, which included 4 items on family impact. It was then field-tested with a convenience sample of parents/caregivers of 5-year-olds. The ECOHIS has a family impact component, but the FIS has been shown to be better for health services research work, such as assessing the improvement in family impact which is observed after children with early childhood caries are treated under general anaesthetic [9]. There is also the issue of the absence from the ECOHIS of an item pertaining to disrupted sleep, an impact which is common among families of children with severe early childhood caries (ECC). Thus, the ECOHIS falls short in respect of some important features.

Accurate and valid measurement of the family impact of such conditions might be better achieved, then, by using the FIS-8 (or both measures concurrently). However, there is no published information on the concurrent validity and reliability of the FIS-8 in a US child population. Accordingly, the aim of this study was to examine those aspects of the FIS-8 in a sample of four-year-old US children taking part in a large multi-center cohort study.

## Methods

This prospective longitudinal study was managed and coordinated at the University of Michigan (Ann Arbor, MI). Three well-established primary care medical research networks—the Pediatric Research Network (Indiana University) in Indianapolis, IN; the Iowa Research Network (Iowa University) in Iowa City, IA, and Duke University’s Primary Care Practice-Based Research Network in Durham, NC—identified and enrolled 1,326 children (age at baseline visit: 12 ± 3 months old). The study population was stratified by Medicaid status, and the sample was intended to be diverse in relation to racial/ethnic group and urban/rural residence. Child-caregiver dyads were identified for possible recruitment primarily through well-child medical appointments, but other venues and approaches were also used (such as neighborhood centers, daycare centers, and advertising [10, 11]). The site teams additionally facilitated two follow-up in-person visits at child ages 30 ± 3 months (2.5 years after baseline; 80% follow-up, or *N* = 1,060) and 48 ± 3 months (4 years after baseline; 74% follow-up, or *N* = 982). To enhance retention and check for access to care and preventive services, intermediate contacts (that is, by phone, mail, email, etc.) occurred every four months throughout the study, with a postcard sent every birthday.

The impact of the child’s oral condition on the family was measured at age 48 months, with data from a subsample of 422 caregivers (of a total of 686 who had been approached). Institutional Review Board approval for the portion of these assessments done at Duke University took longer than anticipated [12], and so Duke site families are under-represented in the current study.

The assessment of family impact using the FIS-8 [3] was included as part of the parental questionnaire. The FIS-8 uses a five point Likert-like scale, with response options “Never” (scoring 0), “Once or twice” (1), “Sometimes” (2), “Often” (3) and “Every day or almost every day” (4). An overall score (range 0 to 32, with higher scores reflecting greater family impact of the child's oral condition) was computed by summing the scores for all items, after which the three subscale scores were computed.

Two global items with ordinal response scales were used for examining the concurrent validity of the FIS-8. The first was the global family impact item “How much are your family’s daily lives affected by your child’s teeth, lips, jaws or mouth?”. This used the response options ‘Not at all’ (scoring 1), ‘Very little’ (2), ‘Some’ (3), ‘A lot’ (4) and ‘Very much’ (5). The second was the global oral health item “How would you describe the health of your child’s teeth and mouth?”, with response options ‘Excellent’ (scoring 1), ‘Very good’ (2), ‘Good’ (3), ‘Fair’ (4) and ‘Poor’ (5).

Clinical examinations of the children at age 4 were undertaken using International Caries Detection and Assessment System (ICDAS) criteria [13]. Children’s teeth were brushed before examination by trained and calibrated examiners who used a mirror and ball-ended probe (for confirmation of ICDAS lesions, where appropriate). For the current paper, only caries lesions at the d3 level (localised enamel breakdown) or higher (definite dentin involvement) are reported.

### Statistical analyses

First, internal consistency reliability was assessed using Cronbach’s alpha (for the subscales and overall scales), with values of 0.7 and more considered acceptable. Summated scale scores then were computed. Concurrent validity then was determined by examining the gradients in mean FIS-8 scale and subscale scores across the ordinal categories of the global ratings of overall family impact and child oral health; Spearman correlation coefficients were also calculated, to complement the information from those gradients. The skewed distributions of the scale scores meant that non-parametric statistics were used (Mann–Whitney U or Kruskal–Wallis tests, as appropriate) to determine the statistical significance (with an alpha value of 0.05) of the observed differences. Sociodemographic and other differences in scale scores were examined using Mann–Whitney or Kruskal–Wallis tests, as appropriate for the number of categories of the independent variable.

## Results

Family impact scale data were collected at child age 48 months from a subset of study participants (*N* = 422), comprising 31.8% of the full sample. Of the 422 in the current analysis, 44 (10.4%) were in the Duke University sample, 179 (42.4%) in the Indiana University sample, and 199 (47.2%) in the University of Iowa sample. There were some systematic differences between the 422 in the current analysis and the other participants, whereby White children and tertiary-educated parents were over-represented in the former (Table 1). Similarly, the University of Iowa sample contributed disproportionately to the current study’s sample.

**Table 1:** Comparison of those for whom FIS-8 data were available and those for whom it was not (brackets contain column % unless otherwise indicated)

	FIS-8 data not available	FIS-8 data available	Both combined
Number (row %)	903 (68.2)	422 (31.8)	1325 (100.0)
Sex			
Male	456 (50.5)	220 (52.1)	676 (51.0)
Female	447 (49.5)	202 (47.9)	649 (49.0)
Race of child			
White	346 (38.3)	253 (60.0)^a^	599 (45.2)
Black	388 (43.0)	118 (28.0)	506 (38.2)
Other	169 (18.7)	51 (12.1)	220 (16.6)
Parental education			
Up to high school	185 (20.5)	90 (21.3)^b^	275 (20.8)
Some college	284 (31.5)	228 (54.0)	512 (38.6)
Masters or above	105 (11.6)	92 (21.8)	197 (14.9)
Unknown	329 (36.4)	12 (2.8)	341 (25.7)
Site			
Duke University	390 (43.2)	44 (10.4)^c^	434 (32.8)
Indiana University	364 (40.3)	179 (42.4)	543 (41.0)
University of Iowa	149 (16.5)	199 (47.2)	348 (26.3)

^a^*P* < 0.001; df = 2; Chi-square = 54.35

^b^*P* < 0.001; df = 3; Chi-square = 184.15

^c^*P* < 0.001; df = 2; Chi-square = 214.16

For the purposes of the subsequent analyses, those in the ‘Unknown’ category for parental education were combined with the ‘Up to high school’ category.

Summary data on the dental caries experience of the sample are presented in Table 2 by sociodemographic characteristics and responses to the global items. Just over one in five had one or more d3mft, with that proportion being higher among children whose parents had not attended college, those from homes where English was not spoken, and those without an employed adult in the home. There were similar patterns—as well as an ethnic difference—with the mean d3mft. Children whose oral health was rated as worse on the global items had higher d3mft scores, on average, and greater proportions of them had had dental caries experience.

**Table 2:** Summary data on dental caries experience by sociodemographic characteristics and responses to global items (brackets contain row percentages unless otherwise indicated)

	Number of children with 1 + d3mft^a^	Mean d3mft score (sd)^a^
*Sociodemographic characteristics*		
Sex		
Male	47 (22.3)	0.7 (1.9)
Female	36 (18.3)	0.7 (2.0)
Race of child		
White	11 (28.9)	0.4 (1.4)^g^
Black	3 (50.0)	1.0 (2.0)
Other	69 (19.0)	1.3 (2.9)
Parental education		
Up to high school	33 (37.5)^b^	1.6 (2.9)^h^
Some college	38 (16.7)	0.5 (1.4)
Masters or above	12 (13.0)	0.3 (0.8)
Language spoken at home		
English	64 (18.4)^c^	0.6 (1.6)^i^
Other	19 (31.7)	1.2 (2.7)
Employed adult in home		
No	15 (38.5)^d^	1.4 (2.6)^j^
Yes	68 (18.4)	0.6 (1.7)
*Responses to global items*		
Health of child’s teeth or mouth		
Excellent	11 (7.7)^e^	0.3 (1.2)^k^
Very good	29 (17.0)	0.4 (1.4)
Good	27 (37.5)	1.5 (2.8)
Fair	13 (72.2)	3.6 (3.2)
Poor	2 (100.0)	5.5 (0.7)
How much are your family’s daily lives affected by child’s teeth, lips, jaws or mouth?		
Not at all	47 (16.4)^f^	0.6 (1.7)^l^
Very little	24 (25.5)	0.9 (2.2)
Some	9 (52.9)	2.2 (2.8)
A lot	1 (14.3)	0.1 (0.4)
Very much	1 (33.3)	3.3 (5.8)
All combined	83 (20.3)	0.7 (1.8)

^a^One or more teeth with cavitated caries lesions; caries experience data missing for 14 children

^b^*P* < 0.001; df = 3; Chi-square = 20.13

^c^*P* = 0.03; df = 1; Chi-square = 5.53

^d^*P* = 0.02; df = 1; Chi-square = 5.35

^e^*P* < 0.001; df = 4; Chi-square = 66.49

^f^*P* = 0.003; df = 4; Chi-square = 15.98

^g^*P* = 0.003; df = 2; Kruskal–Wallis H = 22.91

^h^*P* < 0.001; df = 2; Kruskal–Wallis H = 22.29

^i^*P* = 0.03; df = 1; Mann–Whitney U test; Z = -2.14

^j^*P* = 0.02; df = 1; Mann–Whitney U test; Z = -2.4

^k^*P* < 0.001; df = 4; Kruskal–Wallis H = 74.84

^l^*P* = 0.001; df = 4; Kruskal–Wallis H = 17.61

Data on responses to the individual FIS-8 items are presented in Table 3. Overall, responses were positively skewed, with more than 80% of responses to any given item being ‘Never’, and very small proportions responding ‘Often’ or ‘Every day’.

**Table 3:** Responses to individual FIS-8 items (data are the number of responses; brackets contain row %)

	Never	Once/twice	Sometimes	Often	Every day
Felt guilty	359 (85.1)	41 (9.7)	17 (4.0)	4 (0.9)	1 (0.2)
Been upset	343 (81.3)	48 (11.4)	25 (5.9)	4 (0.9)	2 (0.5)
Had sleep disrupted	360 (85.3)	32 (7.6)	21 (5.0)	4 (0.9)	5 (1.2)
Required more attention from you or others in the family	343 (81.3)	38 (9.0)	31 (7.3)	8 (1.9)	2 (0.5)
Taken time off work	365 (86.5)	41 (9.7)	12 (2.8)	3 (0.7)	1 (0.2)
Had less time for yourself or the family	370 (87.7)	31 (7.3)	14 (3.3)	3 (0.7)	4 (0.9)
Blamed you or another person in the family	384 (91.0)	24 (5.7)	12 (2.8)	1 (0.2)	1 (0.2)
Argued with you or others in the family	371 (87.9)	37 (8.8)	12 (2.8)	1 (0.2)	1 (0.2)

Factor analysis (using the principal components method) revealed two components, respectively representing 45.7% (eigenvalue 3.7) and 15.0% (eigenvalue 1.2) of the variance in responses. Cronbach’s alpha for the overall FIS-8 was 0.83. For the parental emotions subscale, it was 0.43, and it was 0.78 for the parental/family activity subscale and 0.64 for the family conflict subscale.

Data on the concurrent validity of the FIS-8 and subscales are presented in Table 4. Gradients in mean scores across the ordinal response categories of the global family impact item were inconsistent, with mean scores highest among those responding ‘Some’ to that item. By contrast, there were marked and consistent gradients in mean scores across the ordinal categories of the global item on the child’s oral health, with scores highest for those rating their child’s oral health as ‘Poor’. Despite those marked gradients, the correlations were weak.

**Table 4:** Mean FIS-8 and subscale scores by global item responses (brackets contain standard deviations unless otherwise indicated)

			Mean FIS-8 subscale scores		
	Number responding (%)	Mean FIS-8	Parental emotions	Parental/family activity	Family conflict
How much are your family’s daily lives affected by your child’s teeth, lips, jaws or mouth?^a^					
Not at all	297 (70.5)	1.2 (2.7)^b^	0.3 (0.7)^b^	0.7 (1.8)^b^	0.2 (0.7)^b^
Very little	97 (23.0)	2.2 (3.3)	0.7 (1.1)	1.1 (2.0)	0.4 (0.8)
Some	17 (4.0)	6.1 (5.6)	1.8 (2.0)	3.0 (3.5)	1.3 (1.6)
A lot	7 (1.7)	5.1 (6.5)	1.4 (1.9)	2.7 (3.0)	1.0 (1.7)
Very much	3 (0.7)	3.0 (1.0)	1.3 (1.5)	1.7 (2.1)	0.0 (0.0)
Spearman correlation		0.21	0.22	0.11	0.19
How would you describe the health of your child’s teeth and mouth?^f^					
Excellent	147 (35.0)	1.1 (2.6)^b^	0.3 (0.8)^b^	0.7 (1.8)^b^	0.2 (0.6)^b^
Very good	176 (41.9)	1.5 (2.9)	0.5 (0.9)	0.9 (1.9)	0.2 (0.7)
Good	76 (18.1)	2.1 (3.5)	0.6 (1.0)	1.0 (2.0)	0.4 (1.0)
Fair	19 (4.5)	4.6 (4.8)	1.6 (1.5)	2.1 (3.1)	0.8 (1.1)
Poor	2 (0.5)	12.0 (7.1)	4.0 (2.8)	5.0 (2.8)	3.0 (1.4)
Spearman correlation		0.28	0.23	0.22	0.22
All combined	422 (100.0)	1.7 (3.2)	0.5 (1.0)	0.9 (2.0)	0.3 (0.8)

^a^One missing response

^b^*P* < 0.001; df = 4; Kruskal–Wallis H = 36.08

^c^*P* < 0.001; df = 4; Kruskal–Wallis H = 26.68

^d^*P* < 0.001; df = 4; Kruskal–Wallis H = 28.00

^e^*P* < 0.001; df = 4; Kruskal–Wallis H = 29.00

^f^Two missing responses

^g^*P* < 0.001; df = 4; Kruskal–Wallis H = 23.87

^h^*P* < 0.001; df = 4; Kruskal–Wallis H = 32.60

^i^*P* = 0.02; df = 4; Kruskal–Wallis H = 12.29

^j^*P* < 0.001; df = 4; Kruskal–Wallis H = 34.91

Mean FIS-8 and subscale scores are presented by sociodemographic characteristics in Table 5. There were no apparent sex differences. Black children had higher scores, on average. There were consistent gradients by parental education level, whereby mean scores were highest for those with the least education. Mean scores were also higher for homes in which no adult was employed.

**Table 5:** FIS-8 scores by sociodemographic characteristics

		Mean FIS-8 subscale scores		
	Mean FIS-8	Parental emotions	Parental/family activity	Family conflict
Sex				
Male	1.5 (2.7)	0.4 (0.9)	0.8 (1.8)	0.3 (0.7)
Female	2.0 (3.7)	0.6 (1.1)	1.1 (2.3)	0.3 (0.9)
Race of child				
White	1.1 (2.4)^a^	0.4 (0.9)^b^	0.5 (1.3)^c^	0.2 (0.7)^d^
Black	3.2 (4.3)	0.7 (1.2)	1.9 (2.8)	0.5 (1.0)
Other	1.8 (3.1)	0.5 (1.0)	1.0 (1.9)	0.3 (0.8)
Parental education				
Up to high school	3.1 (4.4)^e^	0.8 (1.3)^f^	1.8 (2.8)^g^	0.5 (1.0)^h^
Some college	1.5 (3.0)	0.4 (0.9)	0.8 (2.0)	0.3 (0.8)
Masters or above	1.0 (1.7)	0.4 (0.8)	0.4 (0.7)	0.2 (0.7)
Language spoken at home				
English	1.7 (3.3)	0.5 (1.0)	0.9 (2.0)	0.9 (2.0)
Other	1.8 (3.2)	0.4 (0.8)	1.2 (2.3)	1.2 (2.3)
Employed adult in home				
No	3.2 (4.3)^i^	0.9 (1.5)	1.8 (2.8)	0.3 (0.8)
Yes	1.6 (3.1)	0.5 (0.9)	0.9 (1.9)	0.2 (0.6)

^a^*P* < 0.001; df = 2; Kruskal–Wallis H = 24.39

^b^*P* < 0.001; df = 2; Kruskal–Wallis H = 8.67

^c^*P* < 0.001; df = 2; Kruskal–Wallis H = 31.76

^d^*P* < 0.001; df = 2; Kruskal–Wallis H = 12.05

^e^*P* = 0.01; df = 2; Kruskal–Wallis H = 9.08

^f^*P* = 0.02; df = 2; Kruskal–Wallis H = 7.45

^g^*P* = 0.003; df = 2; Kruskal–Wallis H = 11.88

^h^*P* = 0.02; df = 2; Kruskal–Wallis H = 7.86

^i^*P* = 0.03; df = 1; Mann–Whitney U test; Z = -2.2

## Discussion

This study set out to investigate the concurrent validity and reliability of the Family Impact Scale in a sample of four-year-old US children taking part in a large multi-center cohort study. These were found to be acceptable, as indicated by a Cronbach’s alpha score over the 0.7 threshold [14] for the overall scale (notwithstanding the relatively low score for one of the subscales, most likely arising from it comprising only two items) and the gradients in mean scores across two global measures being largely consistent and as expected.

An issue with the findings is that the observed impact was generally low. This is reflected in the FIS data, where considerable floor effects were apparent; that is, a relatively high proportion of respondents (59% for the overall scale, and 73%, 69% and 85% for the parental emotions, parental/family activity and family conflict subscales, respectively) had the minimum value possible. By contrast, the typical proportion observed with the minimum value for the FIS in clinical samples of 3- to 6-year-olds awaiting dental rehabilitation under general anaesthetic is of the order of 6% to 10% [4, 5]. Floor effects mean that the scale would not be unlikely to reflect any improvement as a result of any intervention designed to improve the child’s oral health and reduce the associated impact on the family [15]. It may be that, even by four years of age in this sample, there would not have been sufficient time for marked, symptomatic dental caries experience to develop— at least, in most of the children—and affect the oral-health-related quality of life of the child and family. Recently reported summary data on dental caries experience in the children show that only one in five had cavitated carious lesions [12].

The study has some other weaknesses. The factor analysis did not confirm the expected factor structure, but it is important to bear in mind that the original FIS arose from theory rather than empirical analysis [1], and that the FIS-8 was then produced using item impact analysis methods [3]. Given the relatively low levels of impact observed in this US sample, we were not likely to be able to confirm the theory-derived scale structure of the scale, and that turned out to be the case. Further work is needed with US samples with greater disease experience. Moreover, FIS data were available for only a subsample of the larger study sample, and there were some important differences between those with FIS data and those without it. Most notably, minority children and the Duke sample were under-represented (and the predominantly rural Iowa sample over-represented), and this may have been partly responsible for the overall lack of family impact observed. A strength of the study is its recruitment of families from particular communities through purposive over-sampling. For example, the Indiana sample focused on African American parent-infant dyads, while the Iowa one recruited from rural communities. Another strength is that the parent study is a prospective one, meaning that follow-up data to at least age 9 will eventually be available, enabling the examination of longitudinal changes in family impact.

The sociodemographic patterns in FIS-8 scores were informative. That there were no apparent sex differences is largely to be expected, given that the sample was too young for any of the gender-specific differences in chronic disease experience to have yet emerged. The higher mean scores among Black children most likely reflect the socio-economic position differences, given that mean scores were highest for those with the least education and for those from homes in which no adult was employed. These differences supply some indirect support for the scale’s validity.

The FIS-8 has had its validity demonstrated in other populations and cultures, such as Oman [6], New Zealand [3], Libya [16], England [17] and Saudi Arabia [18]. Those studies have all confirmed the scale’s validity, with marked gradients observed in mean scale scores across the ordinal categories of the global “gold standard” item(s) used. Those studies have all involved clinical samples of children undergoing treatment for severe early childhood caries, though, and data from population samples are scarce. This makes the current study’s findings important, because they are a rare examination of FIS-8 validity in a nonclinical sample.

While the study provides a degree of evidence for the validity and internal consistency reliability of the FIS in a U.S. child sample, its replication in samples of older preschoolers with greater disease experience would be informative and useful. Future work with this cohort will document changes in family impact as the children age.

## Conclusions

1. The Family Impact Scale appears to be appropriate for use with US children.

2. More widespread use of the scale—both in the US and internationally— would be helpful in furthering global understanding of the impact of children’s conditions on their households.

## Data Availability

The datasets used and/or analysed during the current study are available from MF on reasonable request.

## Abbreviations

FIS-8: Short-form Family Impact Scale
COHQOL: Child Oral Health Quality of Life questionnaire
ECOHIS: Early Childhood Oral Health Impact Scale
ICDAS: International Caries Detection and Assessment System
